# *In vitro* inhibitory effects of plant-derived by-products against *Cryptosporidium parvum*

**DOI:** 10.1051/parasite/2016050

**Published:** 2016-09-14

**Authors:** Klaus Teichmann, Maxime Kuliberda, Gerd Schatzmayr, Thomas Pacher, Karin Zitterl-Eglseer, Anja Joachim, Franz Hadacek

**Affiliations:** 1 Department of Microbiology and Ecosystem Science, University of Vienna Althanstraße 14 1090 Vienna Austria; 2 BIOMIN Research Center Technopark 1 3430 Tulln Austria; 3 Institute of Animal Nutrition and Functional Plant Compounds, Department for Farm Animals and Veterinary Public Health, University of Veterinary Medicine Vienna Veterinärplatz 1 1210 Vienna Austria; 4 Institute of Parasitology, Department of Pathobiology, University of Veterinary Medicine Vienna Veterinärplatz 1 1210 Vienna Austria; 5 Plant Biochemistry, Albrecht-von-Haller Institute, University of Göttingen Justus-von-Liebig-Weg 11 37077 Göttingen Germany

**Keywords:** *Cryptosporidium*, *In vitro*, Cell culture, Phytogenic, *Olea europaea*, By-product

## Abstract

Disposal of organic plant wastes and by-products from the food or pharmaceutical industries usually involves high costs. In the present study, 42 samples derived from such by-products were screened *in vitro* against *Cryptosporidium parvum*, a protozoan parasite that may contaminate drinking water and cause diarrhoea. The novel bioassay was previously established in the microtitre plate format. Human ileocaecal adenocarcinoma (HCT-8) cell cultures were seeded with *C. parvum* oocysts and parasite development was monitored by an indirect fluorescent antibody technique (IFAT) and microscopic assessment for clusters of secondary infection (CSI). Minimum inhibitory concentrations (MICs) and potential detrimental effects on the host cells were determined. An ethanolic extract from olive (*Olea europaea*) pomace, after oil pressing and phenol recovery, reproducibly inhibited *C. parvum* development (MIC = 250–500 μg mL^−1^, IC_50_ = 361 (279–438) μg mL^−1^, IC_90_ = 467 (398–615) μg mL^−1^). Accordingly, tyrosol, hydroxytyrosol, *trans-*coniferyl alcohol and oleuropein were selected as reference test compounds, but their contributions to the observed activity of the olive pomace extract were insignificant. The established test system proved to be a fast and efficient assay for identifying anti-cryptosporidial activities in biological waste material and comparison with selected reference compounds.

## Abstract (fr)

L’élimination des déchets végétaux organiques et des sous-produits des industries alimentaires ou pharmaceutiques invoque généralement des coûts élevés. Dans la présente étude, 42 échantillons dérivés de ces sous-produits ont été testés *in vitro* contre *Cryptosporidium parvum*, un protozoaire parasite provoquant des contaminations de l’eau potable et des diarrhées. Le bioessai nouveau a été établi précédemment sous la forme de plaques de microtitrage. Des cultures de cellules humaines d’adénocarcinome iléocæcal (HCT-8) ont été ensemencées avec des oocystes de *C. parvum* et le développement du parasite a été suivi par technique d’immunofluorescence indirecte (IFAT) et évaluation microscopique pour les foyers d’infection secondaire (CSI). Les concentrations minimales inhibitrices (CMI) et les effets néfastes potentiels sur les cellules hôtes ont été déterminés. Un extrait éthanolique de grignons d’olives (*Olea europaea*), après extraction de l’huile et récupération du phénol, a inhibé de manière reproductible le développement de *C. parvum* (CMI = 250–500 μg mL^−1^, IC_50_ = 361 (279–438) μg mL^−1^, IC_90_ = 467 (398–615) μg mL^−1^). En conséquence, le tyrosol, l’hydroxytyrosol, l’alcool trans-coniféryl et l’oleuropéine ont été choisis comme composés pour des essais de référence, mais leurs contributions à l’activité observée de l’extrait de grignons d’olive étaient insignifiantes. Le système de test établi a prouvé qu’il était rapide et efficace pour identifier les activités anticryptosporidiales dans du matériel issu de déchets biologiques et pour des comparaisons avec des composés de référence sélectionnés.

## Introduction

*Cryptosporidium parvum* is a protozoan parasite of worldwide distribution with significance for both human and animal health [33]. Cryptosporidiosis is characterised by transient diarrhoea and associated problems like malabsorption and dehydration, and can follow a severe course in immunocompromised patients and young animals. Cryptosporidia are naturally resistant to many drugs with known anti-protozoal activities. Despite a large-scale screening experiment indicating some activity for 40 out of 101 tested drugs [46], only a few were able to suppress parasite development completely at low concentrations *in vitro*. Consequently, availability of anti-cryptosporidial drugs for treatment of affected patients is still extremely limited. Azithromycin, paromomycin and nitazoxanide are mostly used, together with other strategies like highly active anti-retroviral therapy (HAART) [38]. Likewise, only a small number of drugs have been found to be effective against animal cryptosporidiosis [33].

Plants present a rich source of bioactive compounds and have a long history of use for prevention and treatment of various human and animal illnesses, including parasitic diseases [1, 20, 29, 31]. For example, the isoflavone genistein from soybean, the flavonolignan mix silymarin (with silibinin as a main compound) from the milk thistle [22, 39, 43] or the ferulic acid-derived curcuminoid curcumin from turmeric [34] have been found to possess anti-cryptosporidial activity *in vitro*. The xanthone mangiferin (known from mango) has demonstrated some efficacy *in vivo* [30]. Recently, pomegranate peel powder was shown to effectively counteract an experimental *Cryptosporidium parvum* infection in mice [3], and a pomegranate extract has shown potential to alleviate *Cryptosporidium*-associated morbidity in calves [45].

This general framework prompted the investigation of the potential effects of selected wastes and by-products against *C. parvum.* Avoiding waste production and re-use of waste have become essential in the society of today and tomorrow, as stated for example in the United Nations Environment Programme’s Agenda 21 [44]. The worldwide annual amount of organic waste and by-products generated is difficult to estimate, due to poor data availability and differing or overlapping definitions of “waste” and “by-products”. According to Eurostat [15], in 2010 more than 2400 million tons of waste were registered in the EU member states, of which more than 3% were animal and plant waste (about 80 million tons). Feeding by-products from food production processes to livestock represents an established procedure to reduce waste accumulation and contributes to economically affordable animal products [35].

The EU-funded project SAFEWASTES [14] not only explored the nutritive value of several plant wastes and by-products, but also aimed at identifying potential health benefits for farm animals. Samples were provided by various industry partners such as juice producers, oil mills and pharmaceutical companies processing medicinal plants. A fast and simple technique for *in vitro* screening of phytogenic samples for activity against *C. parvum* in cell cultures was recently developed [43]. The assay was used in the present study to test 42 samples derived from 18 different plant wastes and by-products by extraction with solvents of different polarity (water, aqueous ethanol or heptane). Identification of the active substance was attempted by High pressure liquid chromatography (HPLC) analysis and by testing pure compounds and specific extracts from anti-cryptosporidial samples.

## Materials and methods

### Extraction

Dried raw material of phytogenic by-products was obtained from various sources, mainly from industrial production (Table 1A), and subjected to extraction by solvents of different polarity, deionised water, 70% aqueous ethanol (v/v) or heptane [40]. Dry plant waste material was milled and mixed with the solvent at a ratio of 1:10–1:20 (w/v). The mixture was continuously stirred at room temperature for 2 hr and filtered through a 0.45 μm filter (Phenex^TM^-RC26 mm, Phenomenex, Torrance, CA, USA) or filter paper (Whatman^TM^ folded filters 604½, GE Healthcare, Munich, Germany). The filtered water extracts were immediately lyophilised. The filtered ethanol extracts were evaporated at 30 °C and the remaining liquid was lyophilised. The filtered heptane extracts were evaporated to dryness at 30 °C. The dried extracts were collected and weighed (for yields, see Table 1A). Forty extracts were obtained from 16 different plant species. Grape seed water extract (VVW) and horse chestnut wastewater (AHW) were obtained from the supplier as powder (Table 1A). Samples were stored at room temperature in amber glass bottles, protected from light and under nitrogen atmosphere. Stock solutions for testing were prepared in cell culture medium at a concentration of 4 mg mL^−1^. Dissolving was supported by shaking (circular shaker), heating up to 40 °C or short ultrasonic bath treatment for several seconds. In case of insoluble components, absolute ethanol or dimethyl sulfoxide (DMSO) was added up to 1% (v/v) final concentration in the assay. Stocks were stored at −20 °C and protected from light.

**Table 1.** **Plant by-products for screening for *in vitro* activity against *C. parvum***. List of raw materials and extracts obtained from phytogenic by-products in the course of the EU project SAFEWASTES: (A) extracts and pure compounds related to olives, and (B) monensin. Extracts were abbreviated using the first letters of the genus and the specific epitheton, together with the type of extract produced: W = water extract, E = ethanol extract (70% (v/v)), H = heptane extract. Monensin was used as a positive control. Table according to Stockhammer et al. [40], modified.

Raw material	Scientific name	Product after industrial production process	Origin	Extracts and yields from extraction [g/kg raw material]
(A)				
Horse chestnut wastewater	*Aesculus hippocastanum* L.	Dried wastewater from seeds after methanol-ethanol extraction	Italy	AHW [n.a.^a^]
Hawthorn fruits	*Crataegus monogyna* Jaquin emend. Lindman; *Crataegus laevigata* (Poiret) De Candolle	Dried fruits after ethanol extraction	Austria	CFW [62.6], CFE [52.3]
Pumpkin	*Cucurbita pepo* L. var. Styriaca	Dried, cut and powdered fruits and peels	Austria	CPW [400.0], CPE [275.0], CPH [8.0]
Artichoke	*Cynara scolymus* L. cv. Camus	Dried aerial parts after chaffing, steaming and pressing	Germany	CSW [102.7], CSE [63.7]
Carrot pomace	*Daucus carota* L. cv. Carotan	Dried roots after blanching and pressing	Germany	DCW [220.6], DCE [272.0]
Purple coneflower	*Echinacea purpurea* (L.) Moench	Dried aerial parts after blanching, milling and pressing	Europe	EPW [63.0], EPE [71.6], EPH [3.6]
Sunflower seeds	*Helianthus annuus* L.	Dried seeds after heating and pressing	Argentina	HAW [153.0], HAE [18.5]
Larch sawdust	*Larix decidua* Mill., syn. *Larix europaea* DC	Dried and powdered sawdust	Austria	LDW [34.0], LDE [20.0], LDH [7.0]
Linseed pomace	*Linum usitatissimum* L.	Dried seeds after heating and pressing	Argentina	LUW [190.3], LUE [76.7]
Tomato peels	*Solanum lycopersicum* L.	Dried paste after methanol extraction	Italy	LEW [408.0], LEE [340.0]
Mango peels	*Mangifera indica* L. cv. Kaew	Dried and crushed peels	Thailand	MIW [364.1], MIE [413.5]
Olive pomace	*Olea europaea* L.	Dried fruit pomace after oil pressing and phenol recovery by methanol-ethanol extraction	Italy	OEW [24.0], OEE [20.0]
Willow bark	*Salix alba* L.	Dried bark after ethanol extraction	Germany	SAW [21.6], SAE [24.5], SAH [3.1]
Sinupret	*Primula veris* L., *Primula elatior* L. Hill, *Sambucus nigra* L., *Verbena officinalis* L., *Gentiana lutea* L., *Rumex acetosa* L.	Dried residues after ethanol extraction of primrose, elder and verbena blossoms, gentian leaves and garden sorrel roots	Germany	SIW [14.0] SIE [17.2], SIH [11.0]
Saw palmetto fruits	*Serenoa repens* (Bartram) Small, syn. *Sabal serrulata* (Michaux) Nutall ex Schultes	Dried fruits after extraction with ethanol and methanol (SRH) or after supercritical CO_2_ extraction	USA	SRH [4], SRCW [28.9], SRCE [45.9], SRCH [5.4]
Thyme leaves	*Thymus vulgaris* L.	Dried leaves after ethanol extraction	Germany	TVW [60.2], TVE [70.5], TVH [7.0]
Blueberry peels	*Vaccinium myrtillus* L.	Dried peels after extraction with aqueous alcohols (methanol, ethanol, 2-propanol) and toluene	Italy	VMW [39.5], VME [32.4]
Grape seed extract	*Vitis vinifera* L.	Dry extract obtained by acetone/water and ethyl acetate extraction	Germany	VVW [n.a.^a^]
(B)				
Sample	Purity (%)	Supplier	Order no.	
Hydroxytyrosol from olive leaves	10	Eurochem Feinchemie GmbH, Gröbenzell, Germany	L06C001	
Tyrosol	98	Sigma-Aldrich, St. Louis, MO, USA	188255	
*trans*-Coniferyl alcohol	≥97	Sigma-Aldrich, St. Louis, MO, USA	27740	
Oleuropein extract NATURA from olive leaves	8.2	Sinoplasan AG, Esslingen, Germany	n.a.^a^	
Monensin sodium	90–95	Sigma-Aldrich, St. Louis, MO, USA	M5273	

a not applicable.

### Chemical analysis

Extracts were analysed by HPLC (Waters Corporation, Milford, MA, USA: 626 pump, 600S controller, 717plus autosampler and 996 photodiode array detector with Empower software; column: Phenomenex Luna C18, 150 × 4.6 mm, 5 μm), using a 50 min gradient of acetonitrile (14–35%) in aqueous buffer (1.5 mM tetrabutylammonium hydroxide, 15 mM *o*-phosphoric acid) at a flow rate of 1 mL min^−1^. *Trans-*coniferyl alcohol (≥97%) and tyrosol (98%) (Sigma-Aldrich, St. Louis, MO, USA) and extracts of olive leaves high in hydroxytyrosol (10%; Eurochem Feinchemie GmbH, Gröbenzell, Germany) or oleuropein (8.2%; Sinoplasan AG, Esslingen, Germany) were used as reference substances (Table 1B). Identification criteria were identical retention times and UV spectra (200–400 nm) of corresponding chromatogram peaks of an extract and a reference substance.

### Bioassay

Test compound stock solutions were serially diluted in culture medium (twofold: 31.25–1000 μg mL^−1^). If negative effects on host cell viability were observed or cell proliferation was lower than 75% as determined by the WST-1 assay (Roche Diagnostics GmbH, Vienna, Austria; conducted as described previously [43]), respective lower concentrations were chosen. All assays were performed in duplicate. When activity of a test substance against *C. parvum* was found in the initial assays, it was retested twice in independent trials. Monensin sodium salt (purity 90–95%; Sigma-Aldrich, St. Louis, MO, USA) was included in each trial as a positive control (4.2–133.5 nM). For the negative (infected, but untreated) control, unmodified culture medium was used. If solvents or detergents, e.g. ethanol or DMSO, were used to dissolve a test substance, equal amounts were incorporated into controls as well. Test substances were evaluated by a previously published assay [43], which is based on an indirect fluorescent antibody technique (IFAT) and the foci detection method [36, 37]. Briefly, confluent monolayers of HCT-8 cells in 96-well microtitre plates were infected with chlorine-treated *C. parvum* oocysts before adding the test substance. An inoculum of 2500 oocysts per well was found to produce a maximum number of parasite clusters per area. After incubation for 48 hr (37 °C, 5% CO_2_, humidified air), the microtitre plates were washed with phosphate-buffered saline (PBS), fixed and incubated with anti-*C. parvum* antibody from 4b4 mouse hybridoma cells (University of Hohenheim, Germany) [28] and FITC-conjugated anti-mouse IgG antibody (Sigma-Aldrich, St. Louis, MO, USA). Microtitre plates were evaluated under a fluorescence microscope. A cluster of secondary infection (CSI) was defined as a group of five or more green fluorescent dots of about 3–5 μm diameter, which were located in relatively close vicinity. Each well was checked for presence or absence of cluster formation. The lowest concentration of a sample which completely prevented CSI formation was defined as the minimal inhibitory concentration (MIC). For test substances that showed reproducible inhibition of CSI formation, dose-response curves were established (SigmaPlot 6.1, SPSS Inc., Chicago, IL, USA). The percentage of parasite inhibition in wells containing test substances was calculated in relation to negative control wells, which were defined as 100% parasite development. IC_50_- and IC_90_-values were calculated by probit regression analysis with 95% fiducial limits (SPSS 10.0, SPSS Inc., Chicago, IL, USA) on the basis of evaluating three microscopic fields per well and counting of the CSIs. Only when IC_50_- and IC_90_-values with fiducial limits within the tested concentration range were obtained, was a sample assumed to show significant parasite inhibition.

## Results

**Figure 1.**
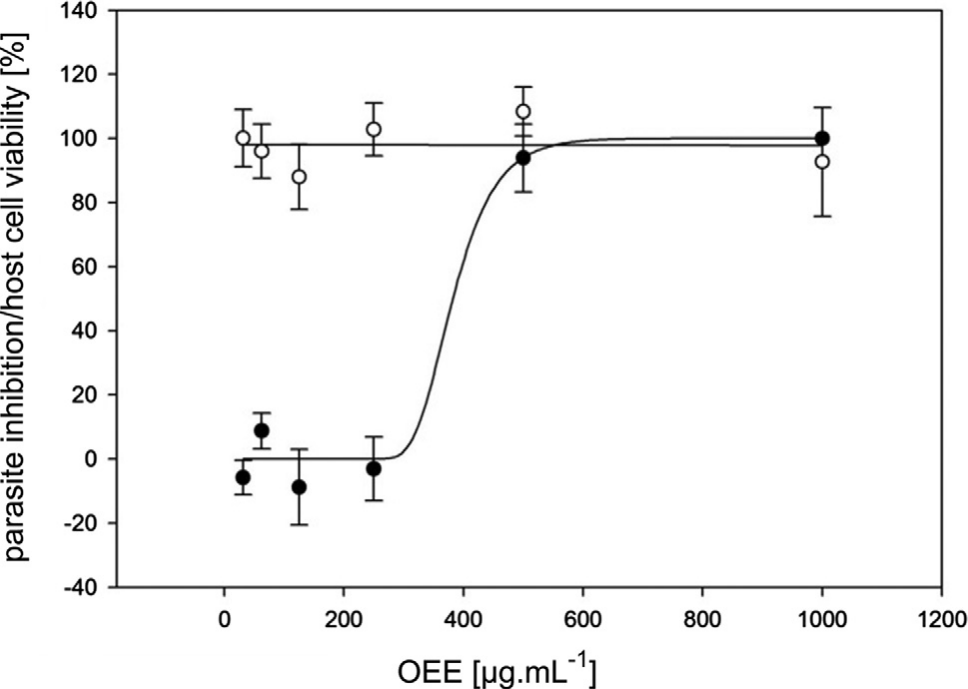
**Olive pomace extract inhibits *Cryptosporidium parvum in vitro***. Dose-response curve for inhibitory activity of an olive ethanolic extract (OEE; from *Olea europaea* L. fruit pomace) against *Cryptosporidium parvum in vitro* (filled dots; *Y*-axis: parasite inhibition) and viability of HCT-8 host cells (empty dots; *Y*-axis: cell viability). Error bars represent the standard error.

In the initial trial, seven out of 42 samples that had been obtained from 18 different plant-derived by-products (Table 1A) completely inhibited *C. parvum* CSI formation within the tested concentration range (≤1000 μg mL^−1^) (Table 2). The complete results table can be found as an online resource (Table S1). Inhibition was only considered as valid if host cell viability remained intact, as determined by the WST-1 assay. Apart from the ethanolic extract from olive pomace (OEE; Fig. 1), all other six initially identified extracts failed to show activity in each of the replication trials. Inhibitory concentrations of OEE were calculated without logarithmic transformation of data: IC_50_ = 361 (279–438) μg mL^−1^, IC_90_ = 467 (398–615) μg mL^−1^ (95% fiducial limits are indicated in brackets). Monensin sodium salt was used as a positive control and showed MICs between 8.3 and 33.4 ng mL^−1^.

**Table 2.** ***In vitro* anti-cryptosporidial activity of plant by-products**. Results from *in vitro* testing of 42 samples derived from 18 different plant by-products, four samples related to olives and monensin sodium salt against *C. parvum*. MIC_100_ indicates the minimal concentration of a sample, at which complete parasite inhibition was observed (μg mL^−1^ for solid samples, nL mL^−1^ for the oleuropein extract or nM for monensin). Samples that were active in the first trial were tested in three independent trials in total, whereas the others were not tested again. Four samples related to olives were tested in two trials, monensin in three. MCC_75_ is the minimal cytotoxic concentration against HCT-8 host cells (<75% host cell viability). Inhibitory concentrations within the non-toxic range for host cells are marked by bold print. The complete results table can be found as an online resource (Table S1).

	MIC_100_			MCC_75_		
Test substance	Trial 1	Trial 2	Trial 3	Trial 1	Trial 2	Trial 3
Horse chestnut wastewater (AHW)	**500**	1000	1000	1000	1000	1000
Olive pomace ethanol extract (OEE)	**250**	**500**	**500**	>1000	>1000	>1000
Willow bark ethanol extract (SAE)	**500**	**250–500**	>500	>1000	>500	>500
Willow bark heptane extract (SAH)	**1000**	**500**	>500	>1000	>500	>500
Sinupret ethanol extract (SIE)	**250–500**	**125–500**	>500	>500	>500	500
Sinupret heptane extract (SIH)	**250**	>500	>500	>500	>500	>500
Grape seed extract (VVW)	**500**	1000	1000	1000	1000	1000
Hydroxytyrosol (10%)	>1000	>1000		>1000	>1000	
Tyrosol (98%)	>250	>250		>250	>250	
Coniferyl alcohol (≥97%)	>250	>250		250	>250	
Oleuropein extract (8.2% oleuropein)	>1000	>1000		>1000	>1000	
Monensin sodium (90–95%)	**16.7**	**16.7–33.4**	**8.3**	>133.5	>133.5	>133.5

**Figure 2.**
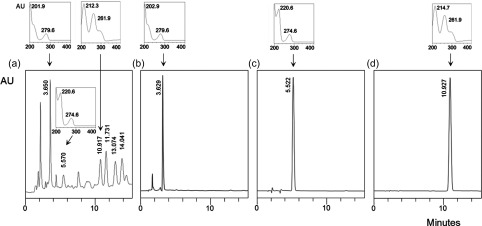
**Chromatography**. HPLC chromatograms and peak spectra of (a) an olive ethanolic extract (OEE; from *Olea europaea* L. fruit pomace; 260 nm) and pure reference compounds which could be confirmed as constituents of the extract, (b) hydroxytyrosol (280 nm), (c) tyrosol (280 nm) and (d) *trans-*coniferyl alcohol (260 nm). Retention times (minutes; rotated view), the relative absorption intensity (AU) and UV spectra together with absorption maxima (wavelengths in nm) are indicated.

HPLC analysis of OEE and selected pure compounds that were expected to occur in an olive extract revealed the presence of tyrosol, hydroxytyrosol and *trans-*coniferyl alcohol in OEE (Fig. 2), while oleuropein was not detected in the extract (data not shown).

The pure reference compounds tyrosol and *trans-*coniferyl alcohol, an oleuropein-rich extract (8.2%) and an extract rich in hydroxytyrosol (10%) failed to display complete parasite inhibition at non-toxic concentrations to the host cells (Table 2).

## Discussion

Poor storage stability of samples or their active compounds may account for the lack of reproducibility of the anti-cryptosporidial activity found for the six initially active samples. Some degree of result variability has been apparent in this assay, expressed by the MIC-range of monensin (8.3–33.4 nM). Moreover, the activity of monensin was slightly higher than those observed in previous experiments [43]. Batch-to-batch-variation and freshness of oocysts may be an additional explanation for the variation in parasite inhibition. Nevertheless, complete inhibition of *C. parvum* at 133.5 nM or lower has been a reliable positive control in these and former experiments.

A dose-response curve for OEE (Fig. 1) showed a steep inclination between the non-inhibitory and the inhibitory concentration ranges. This was also reflected by the narrow distance between the IC_50_ and IC_90_, with 361 and 467 μg mL^−1^, respectively. At the highest tested concentration (1000 μg mL^−1^), host cell viability started declining, indicating slight cytotoxic effects. Thus, the potential therapeutic range of the extract appears to be rather narrow. According to a previous review on the chemical composition of olive fruits [18], water, oil, carbohydrates (including cellulose and pectin), protein, organic acids, pigments and inorganic substances are the main constituents. Additionally, phenols and polyphenols have been identified. Together with unsaturated fatty acids, tocopherols and phospholipids, they are thought to be responsible for a range of health-promoting effects of olives and olive oil [6, 9, 18]. Moreover, phenols are considered essential for the characteristic taste and oxidative stability of olive products. Among the phenolic substances, oleuropein, tyrosol and hydroxytyrosol are highly abundant in olive fruits, their concentrations depending on fruit ripeness [9]. Not all of the phenolic compounds are removed by oil pressing, probably due to their hydrophilic nature. Hydroxytyrosol, oleuropein, tyrosol, caffeic acid, *p*-coumaric acid, vanillic acid, verbascoside, elenolic acid, catechol and rutin were identified as the main phenolic compounds of olive press cake [18].

Consequently, several probable chemical constituents of olive press cake extract were purchased as synthetic or purified chemicals. Comparative HPLC analysis of the OEE indicated the presence of tyrosol, hydroxytyrosol and coniferyl alcohol, but oleuropein was not detectable. This is not surprising, as mature fruits are used for olive oil pressing and oleuropein is known to be degraded to demethyloleuropein, elenolic acid dialdehyde and hydroxytyrosol during the maturation process [4, 9]. Furthermore, *trans-*coniferyl alcohol appeared to be a probable constituent and has not been reported previously from this material. Occurrence of coniferyl alcohol is not surprising, since its derivatives, lignans, have been reported from olive oil [7]. The olive press cake used in the present study had been subjected to a phenol recovery step (methanol-ethanol extraction) by the supplier before extraction for *Cryptosporidium* testing. The detection of simple phenols like tyrosol, hydroxytyrosol and coniferyl alcohol suggests that oil pressing and the phenol recovery step did not remove phenols exhaustively from the olive pomace.

Tyrosol, *trans*-coniferyl alcohol and extracts rich in oleuropein or hydroxytyrosol were tested for anti-cryptosporidial activity in our assay at concentrations resembling or exceeding the active concentration of OEE. However, all of them failed to suppress infection and parasite development at the tested concentrations and therefore might not contribute exclusively to the activity of OEE.

Pentacyclic triterpenes like oleanolic acid and maslinic acid have been reported from olive fruit skin [19]. Maslinic acid has been identified as an anti-protozoal compound inhibiting *Eimeria tenella* in an experimental infection of chicken [13]. Moreover, it showed anti-plasmodial activity *in vitro* [25] and *in vivo* [24] and probably possesses a multitargeted mode of action [26]. Additionally, anti-viral, anti-tumour, anti-inflammatory and anti-oxidant activities have been reported for maslinic acid [13]. This compound might account for the observed activity of OEE and should be given attention in future studies.

The worldwide annual production of virgin olive oil exceeded 3 million tons in 2012 [16], resulting in about 1 million ton of olive press cake, as about one-third of olive fresh mass remains as press cake [18] and more than 30 million cubic metres of olive mill wastewater per year in the Mediterranean region [5], where 95% of the world’s olives are produced [2]. Although spreading olive mill wastewater on agricultural soils and crops and composting of olive press cake may have beneficial effects on the physical properties of soil, it may also pose an environmental problem due to the phytotoxicity of some of its phenolic compounds, preventing its direct use as a fertiliser or compost [5, 21, 27]. Alternative utilisation of olive mill waste would relieve this problem.

Feeding olive oil production residues (wastewater, pomace) to animals is current practice and beneficial due to the high protein content of the pomace. Traditionally, olive pulp has been used in ruminant feed [23]. Investigations of the effects on monogastrics, such as layer chickens, have also been carried out [42]. Beneficial effects on production parameters of fattening pigs have been reported [32]. However, the current study and other research [13, 17, 24] indicate that the material can potentially be useful for prevention of infectious diseases.

As the identification of the anti-cryptosporidial compound(s) from OEE has not been successful to date, further research is required to reveal its identity. If the activity was exerted by different chemicals than those responsible for cytotoxic effects towards the host cells, an activity-guided separation could provide a specific active fraction or pure compound. Moreover, the active substance could also result from chemical transformation processes during olive oil production or phenol recovery. For instance, oxidation and acid hydrolysis can occur during storage of olive oil [8]. Possibly, not one chemical compound is responsible for inhibition of *C. parvum* but rather a synergistic mixture of several substances [41]. Plant metabolites have evolved to possess certain activities depending on the reaction milieu, as described for the pro- and anti-oxidant activities of flavonoids [10–12]. Easily oxidisable compounds, like many anti-oxidants, tend to form radicals that may interfere with essential parasite enzymes; this might represent one conceivable mode of action against *C. parvum* for a material rich in anti-oxidants like OEE.

In summary, OEE has been shown to possess anti-cryptosporidial activity *in vitro*. Further research should identify the active ingredients in OEE to improve understanding of the mode of action and to increase the therapeutic range and efficacy by applying isolated and well-defined active compounds.

## Conflict of interest

The authors declare that they have no conflict of interest.

## Supplementary Material

Anticryptosporidial activity of plant by-products *in vitro*.
