# Supplementary material for: In vitro inhibitory effects of plant-derived by-products against Cryptosporidium parvum
Source: Parasite. 2016 Sep 14;23:41. doi: 10.1051/parasite/2016050 (PMC5028040; doi:10.1051/parasite/2016050)
Supplement: Anticryptosporidial activity of plant by-products in vitro. [file parasite-23-41-s1.pdf]

SUPPLEMENTARY MATERIAL

Table S1. Anticryptosporidial activity of plant by-products *in vitro.*

Results from *in vitro* testing of 42 samples derived from 18 different plant by-products, four samples related to olives, and monensin sodium salt against *C. parvum*. MIC_{100} indicates the minimal concentration of a sample, at which complete parasite inhibition was observed (µg mL^{-1} for solid samples, nL mL^{-1} for the oleuropein extract or nM for monensin). Samples which were active in the first trial were tested in three independent trials in total, whereas the others were not tested again. Four samples related to olives were tested in two trials, monensin in three. MCC_{75} is the minimal cytotoxic concentration against HCT-8 host cells (< 75% host cell viability). Inhibitory concentrations within the non-toxic range for host cells are marked by **bold print.**

	MIC_{100}			MCC_{75}		
**Abbreviation**	**Trial 1**	**Trial 2**	**Trial 3**	**Trial 1**	**Trial 2**	**Trial 3**
AHW	**500**	1000	1000	1000	1000	1000
CFE	> 1000			> 1000		
CFW	> 1000			> 1000		
CPE	> 1000			> 1000		
CPH	> 1000			> 1000		
CPW	> 1000			> 1000		
CSE	> 1000			> 1000		
CSW	> 1000			> 1000		
DCE	> 1000			> 1000		
DCW	> 1000			> 1000		
EPE	> 500			> 500		
EPH	> 1000			> 1000		
EPW	> 1000			> 1000		
HAE	> 1000			> 1000		
HAW	> 1000			> 1000		
LDE	> 1000			1000		
LDH	> 1000			> 1000		
LDW	> 1000			> 1000		
LEE	> 1000			> 1000		
LEW	> 1000			> 1000		
LUE	> 1000			> 1000		
LUW	> 1000			> 1000		
MIE	> 1000			> 1000		
MIW	> 1000			> 1000		
OEE	**250**	**500**	**500**	> 1000	> 1000	> 1000
OEW	> 1000			> 1000		
SAE	**500**	**250 - 500**	> 500	> 1000	> 500	> 500
SAH	**1000**	**500**	> 500	> 1000	> 500	> 500
SAW	> 1000			> 1000		
SIE	**250 – 500**	**125 - 500**	> 500	> 500	> 500	500
SIH	**250**	> 500	> 500	> 500	> 500	> 500
SIW	> 1000			> 1000		
SRCE	> 1000			> 1000		
SRCH	> 1000			1000		
SRCW	> 1000			> 1000		

	MIC_{100}			MCC_{75}		
**Abbreviation**	**Trial 1**	**Trial 2**	**Trial 3**	**Trial 1**	**Trial 2**	**Trial 3**
SRH	> 1000			> 1000		
TVE	1000			1000		
TVH	> 1000			> 1000		
TVW	> 1000			> 1000		
VME	> 500			> 500		
VMW	> 1000			> 1000		
VVW	**500**	1000	1000	1000	1000	1000
Samples related to olives						
Hydroxytyrosol	> 1000	> 1000		> 1000	> 1000	
Tyrosol	> 250	> 250		> 250	> 250	
Coniferyl alcohol	> 250	> 250		250	> 250	
Oleuropein extract	> 1000	> 1000		> 1000	> 1000	
Monensin sodium	**16.7**	**16.7 - 33.4**	**8.3**	> 133.5	> 133.5	> 133.5
